# Free Treatment with Free Medicine

**Published:** 1914-09-26

**Authors:** 


### The Hospital

The Workers' Newspaper of Administrative Medicine and Institutional Life, Administration, National Insurance and Health.

No. 1475, Vol. LVI. SATURDAY, SEPTEMBER 26, 1914. PRICE ONE PENNY.

### FREE TREATMENT WITH FREE MEDICINE.

From one end of the nation to the other all classes and most individuals have been fired with the desire to do something in the cause for which England and the Empire are fighting to-day. The wonderful unity in feeling and practice which pervades us all has naturally found expression on the part of practitioners and pharmacists, who have offered to provide, wherever practicable, free medical attendance, with medicine and appliances, to those dependants of sailors and soldiers serving with the Colours whose circumstances are such as to render them not unsuitable recipients. The proposal originated appropriately with the British Medical Association and the Pharmaceutical Society of Great Britain, and their generous offer has been gratefully accepted by the Government, who fully recognise the magnitude of the sacrifice which it will entail on the two professions and the expenditure and labour involved for the Association and the Society.

We hope that this scheme will be so organised and worked as to make it fruitful in results for the good of the people and also of the profession. For the people, by keeping it entirely free from every taint of charity, whilst it upholds the doctrine that the medical treatment and the services of the Association and the Society are spontaneous offerings of personal service, not as a charity, but as a privilege. Now that the grants are increased and their payment made direct through the Post Office each week to the dependants of our sailors and soldiers serving with the Colours, our warriors' families are put for the first time in the history of the nation in a position of independence. Each representative of a dependant's family is thus placed upon her honour to uphold its independence during the absence of the dear one, and to maintain the home as each warrior left it. This independence will be supplemented, we hope, by the principal householders throughout the country registering their names with the Lord Mayors, Mayors, Chairmen of County Councils, and other local authorities as Friends of these dependants, each registered householder thereby intimating his or her willingness to act as a friend to one sailor's or soldier's family. In this simple way all contact with the Poor Law, with charity organisations, and with distress committees should be absolutely severed, so far as the dependants of our sailors and soldiers are concerned, because the necessity for relief as such should not arise, providing the Friends—*i.e.*, each of them—do their duty by giving counsel, advice, and personal service as and when required.

It will be seen, therefore, that the medical treatment, and the supply of medicines and appliances through the Prince of Wales's Fund, must be regarded on the one hand as spontaneous personal service, and on the other as the fulfilment of a duty which the nation owes to the children of the State. It is necessary to emphasise this important revolution in practice, so far as the dependants of our sailors and soldiers are concerned, because the upholding of this principle means, after the war, a new and better inter-relation between all classes of the people and a higher standard and better homes for the humblest and poorest among them for the future. We hope, therefore, that the special Sub-Committee appointed to bring this scheme into operation will abandon altogether the idea of local relief committees, because they belong to an old order of things, whereby the population has been demoralised through charity and doles. Now charity and doles in the present circumstances would be unworthy of our nation and opposed to the spirit and feelings of all classes of our people, as well as an insult, as we pointed out last week, to the dependants of the soldiers of the State.

It remains to consider how this spontaneous outpouring of service, rightly regarded as a privilege by practitioners and pharmacists, can be made fruitful in good to the members who render it and to the professions which they represent? The whole nation is one people to-day, and our sailors and soldiers have by their devotion set an example which has gone straight to the heart not only of the nation but of the Empire, so that in God's providence great changes for good cannot fail to result ultimately from the war. The profession of medicine has in the past, in Great Britain at any rate, rendered far too large a volume of eleemosynary service. This has had many results fruitful in good, but it has not seldom proved a cruel tax on individual practitioners and those dependent upon them. Before the war evidence was increasingly forthcoming that the supply of practitioners was becoming far too scanty to meet the needs of the people and the requirements of measures which the central and local governments rightly regard as essentials to the health, the happiness, and the maximum earning power of the population. With the progressive development of the nation the cost of living and the expenses of practice have increased out of all proportion to the earning power of the average practitioner as represented by the fees he has been receiving. It is therefore essential that the profession and those who represent its interests in connection with this scheme should bear in mind that, after the war, the time will have come to put all these professional problems upon a firm business basis, which shall protect the interests of the profession and restore to its members a generous provision in the way of revenue. Such a basis will tend not only to do justice to the men already qualified, but to attract the younger men with scientific instincts and high qualifications.

An ever-increasing number of students will be needed to maintain the medical army which exists to fight disease and bring the maximum of health to every individual throughout the country. By refusing to permit the taint of charity to soil the generous outpouring of personal service by the medical profession to the dependants of our sailors and soldiers during the present war, a great step will be taken on the road which will lead the medical profession to a sound and equitable basis, so far as its work and emoluments are concerned. We hope that in the interests of the independence of all classes of the community, for the sake of the maintenance of the highest standard of efficiency in the administration of our hospitals and everything which appertains to the treatment of the patients, another essential change will be the speedy diminution of eleemosynary service, generally, with its total abolition so far as our greater medical institutions and health and sanitary organisations are concerned.

